# Primary Malignant Mesothelioma of the Tunica Vaginalis: A Case Report and Review of the Literature

**DOI:** 10.7759/cureus.94081

**Published:** 2025-10-07

**Authors:** Amgad Elmadani, Ahmed Farag, Zubair Al-Qassim, Roland England

**Affiliations:** 1 Urology, Kettering General Hospital NHS Trust, Kettering, GBR

**Keywords:** hydrocele, inguinal orchidectomy, malignant mesothelioma, testicular swelling, tunica vaginalis tumor

## Abstract

Primary malignant mesothelioma of the tunica vaginalis is an extremely rare neoplasm, and its diagnosis is often unclear initially due to its nonspecific presentation. We present the case of an 87-year-old gentleman who presented with a left-sided hydrocele that increased in size over seven months, with no associated history of asbestos exposure. On initial ultrasound, he had a benign appearing hydrocele and proceeded to hydrocelectomy, where suspicious lesions were noticed and sent for histopathology. A subsequent radical inguinal orchiectomy was performed. Histopathologic examination revealed malignant mesothelioma, which was positive for calretinin, Wilm's Tumour 1 (WT-1), AE1/AE3, and D2-40 immunohistochemically, from both operations. On postoperative staging, no metastases were seen, and the patient has been disease-free on follow-up after six months. This case illustrates the challenges in reaching the diagnosis of malignant mesothelioma of the tunica vaginalis and emphasizes the importance of timely surgical intervention in the form of radical orchidectomy and histological examination of any uncommon scrotal mass. Ongoing case reporting is needed to guide management, as there are no standard guidelines due to the limited number of reported cases.

## Introduction

Malignant mesothelioma of the tunica vaginalis testis (MTVT) is a tumour of mesothelial origin that is extremely uncommon, representing less than 1% of all mesotheliomas, and usually occurs in late adult life, presenting with non-specific scrotal swelling [1,2]. As the published literature has primarily been limited to case reports and small series since the original report by Barbera and Rubino in 1957, the diagnosis and management are difficult to standardize [2,3]. Asbestos exposure is the most well-established etiological factor for pleural and peritoneal mesothelioma; however, it is more tenuously associated with MTVT and has been inconsistent across cohorts. Other contributors include long-standing hydrocele, prior scrotal surgery (e.g., herniorrhaphy), trauma, or chronic inflammation [1,4,5].

Preoperative diagnosis is challenging. Scrotal ultrasound may reveal a complex hydrocele with hypervascular parietal excrescences or papillary vegetations, findings that should prompt suspicion of MTVT; however, imaging is frequently non-diagnostic, and many cases are first discovered intraoperatively during hydrocelectomy or orchiectomy [6,7]. A definitive diagnosis is based on histopathology, with the demonstration of a mesothelial immunophenotype (e.g., calretinin, Wilm's Tumour 1 (WT-1), D2-40/podoplanin, CK5/6) and the exclusion of mimics, such as metastatic adenocarcinoma, using epithelial markers (e.g., Ber-EP4, MOC-31, carcinoembryonic antigen (CEA)) [8-11].

Given the ultra-rare nature of this disease, prospective trials are not feasible, and management approaches are extracted from historical series and pleural/peritoneal mesothelioma experience. Radical inguinal orchiectomy is the bedrock of local therapy, with some centres promoting wider en bloc resections or testing of nodes in a subset of patients [12,13]. Systemic treatment (platinum-pemetrexed most frequently) and, in modern series, immune checkpoint inhibitors have been administered in more advanced disease based on pleural mesothelioma data, in large part, but high-quality data specific to MTVT are still limited [14-18]. This case report contributes to the literature by detailing an elderly patient who presented with a seemingly benign hydrocele and was found to have MTVT pathologically. It serves as a testament to the importance of histopathological evaluation in the setting of false-negative atypical appearing scrotal collections.

## Case presentation

**Figure 1:**
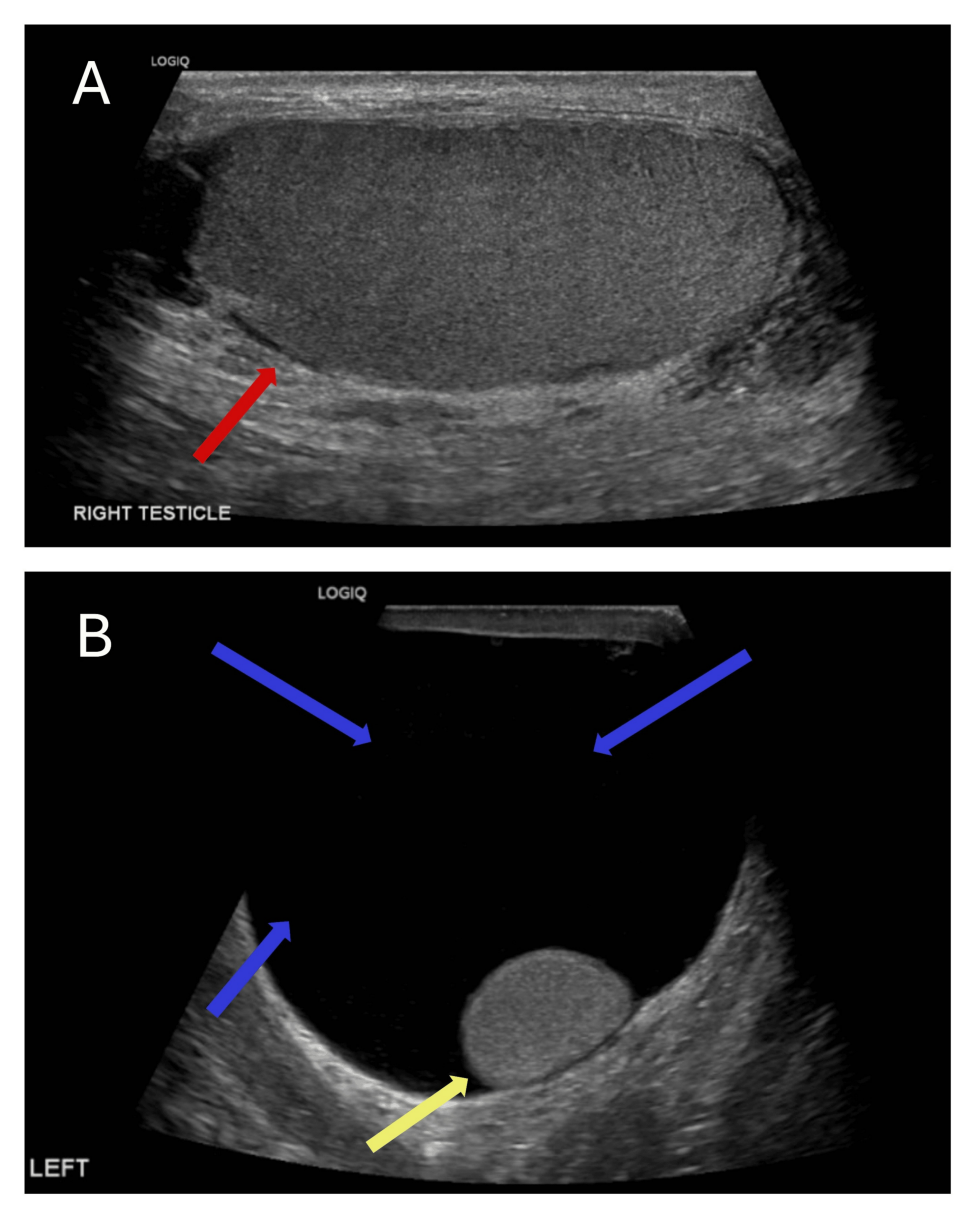
Initial scrotal ultrasound scan showing (A) normal right testicle (red arrow) and (B) a left-sided hydrocele (blue arrows) and a normal left testicle (yellow arrow).

An 87-year-old gentleman, who developed a gradually enlarging left-sided hydrocele over seven months, was referred to our hospital. Physical examination revealed features of a large hydrocele. Scrotal ultrasound demonstrated a large left-sided hydrocele with no sinister features (Figure 1). He reported no history of asbestos exposure.

**Figure 2:**
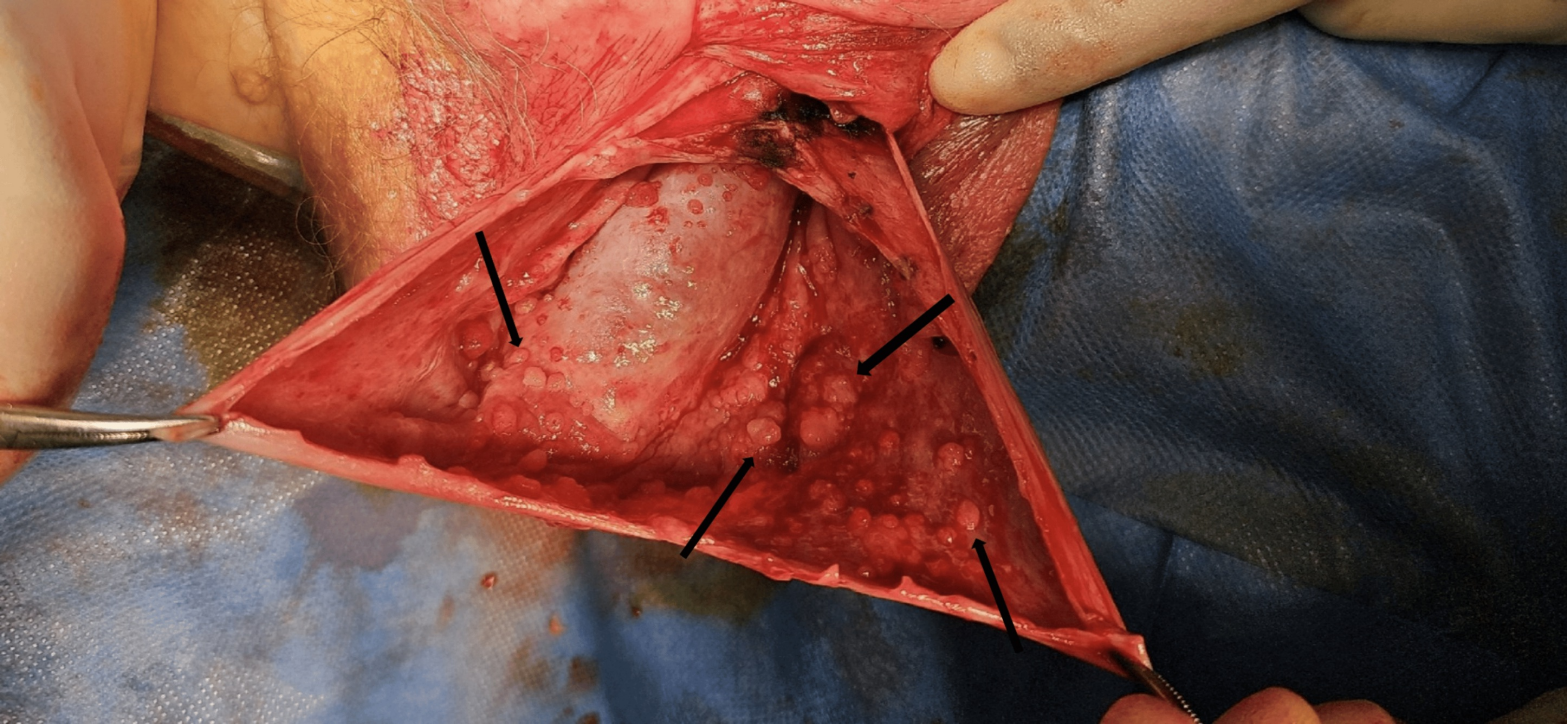
Solid lesions seen within the tunica vaginalis at the time of scrotal exploration (black arrows).

Consequently, the patient was listed for hydrocele repair. Intraoperatively, numerous small solid and cystic masses were identified on the inner surface of the hydrocele sac (tunica vaginalis) (Figure 2). This has raised the suspicion for sinister pathology. Therefore, the decision was made to proceed with hydrocelectomy and wide local excision. The tissue samples were sent for histopathology.

**Figure 3:**
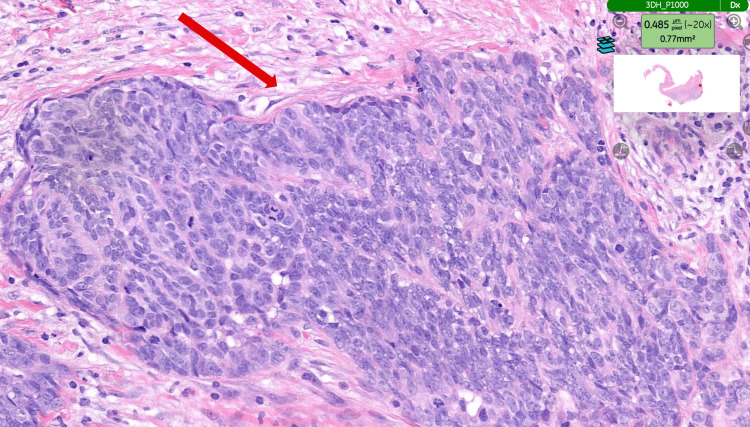
Tumour showing a typical epithelioid pattern. H&E staining. The tissue examined is the hydrocele sac (tunica vaginalis) (magnification ×20). H&E: haematoxylin and eosin.

**Figure 4:**
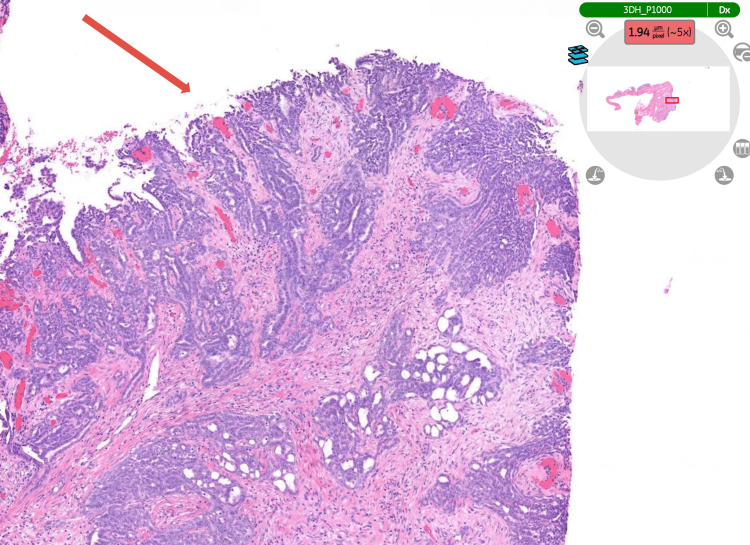
Typical tubulopapillary pattern of mesothelioma. H&E staining. The tissue analysed is the hydrocele sac (tunica vaginalis) (magnification ×5). H&E: haematoxylin and eosin.

**Figure 5:**
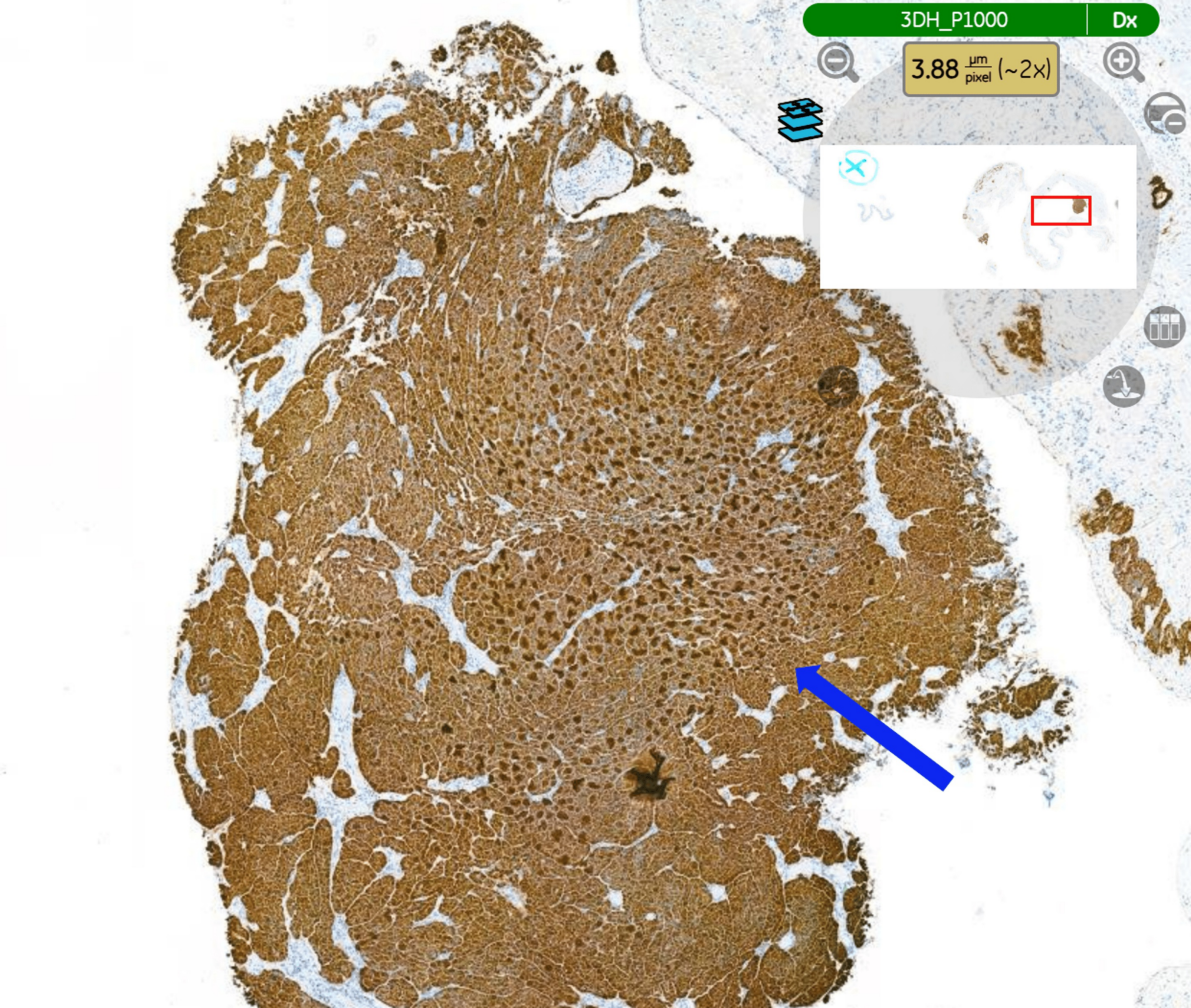
Tumour cells showing diffuse nuclear positivity for calretinin. Immunohistochemical staining (calretinin). The tissue examined is the hydrocele sac (tunica vaginalis) (magnification ×2).

**Figure 6:**
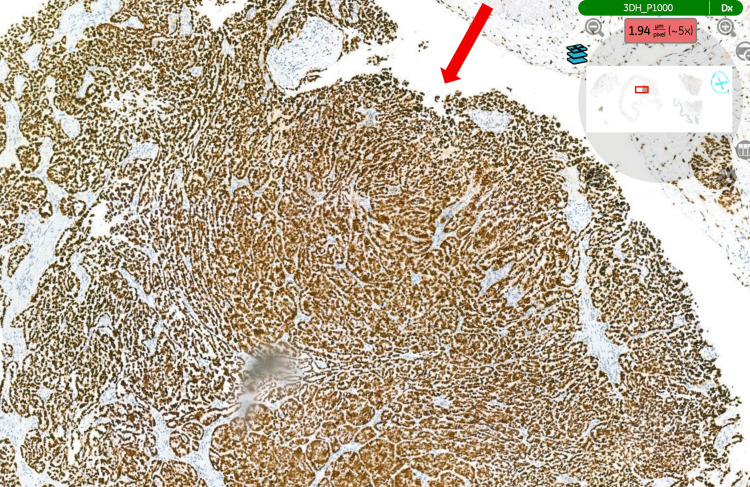
Tumour cells showing diffuse nuclear positivity for WT1. Immunohistochemical staining (WT1). The tissue examined is the hydrocele sac (tunica vaginalis) (magnification ×5). WT1: Wilm's Tumour 1.

**Figure 7:**
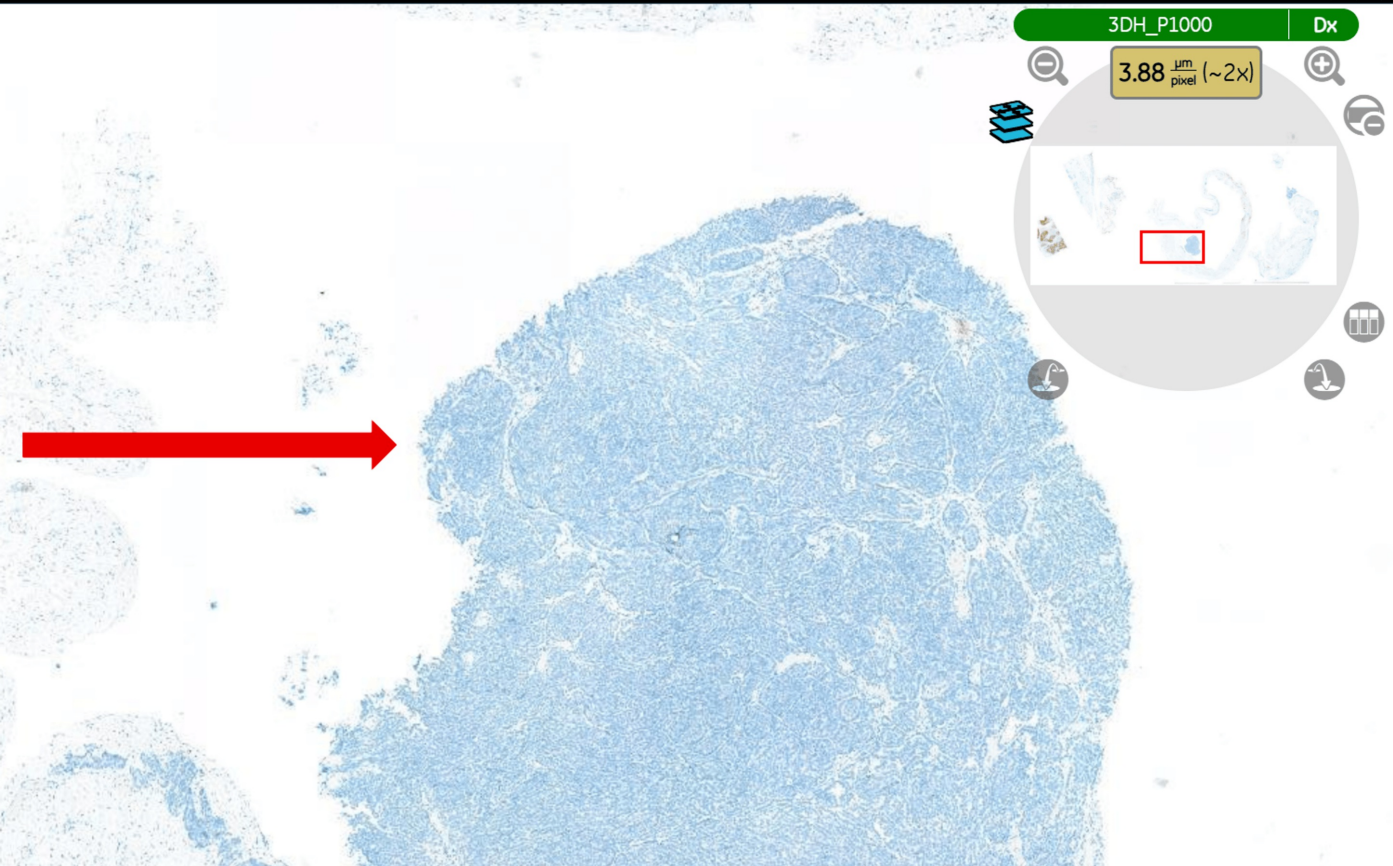
Tumour cells showing negative staining for CEA. Immunohistochemical staining (CEA). The tissue examined is the hydrocele sac (tunica vaginalis) (magnification ×2). CEA: carcinoembryonic antigen.

Histopathological examination of the specimen (hydrocele sac) confirmed an epithelioid malignant mesothelioma of the tunica vaginalis (Figure 3), demonstrating the typical tubulopapillary growth pattern of mesothelioma (Figure 4). Immunohistochemical staining was positive for calretinin (Figure 5), WT-1 (Figure 6), AE1/AE3, and D2-40 and negative for CEA (Figure 7), BER-EP4, CK5/6, and CK20.

**Figure 8:**
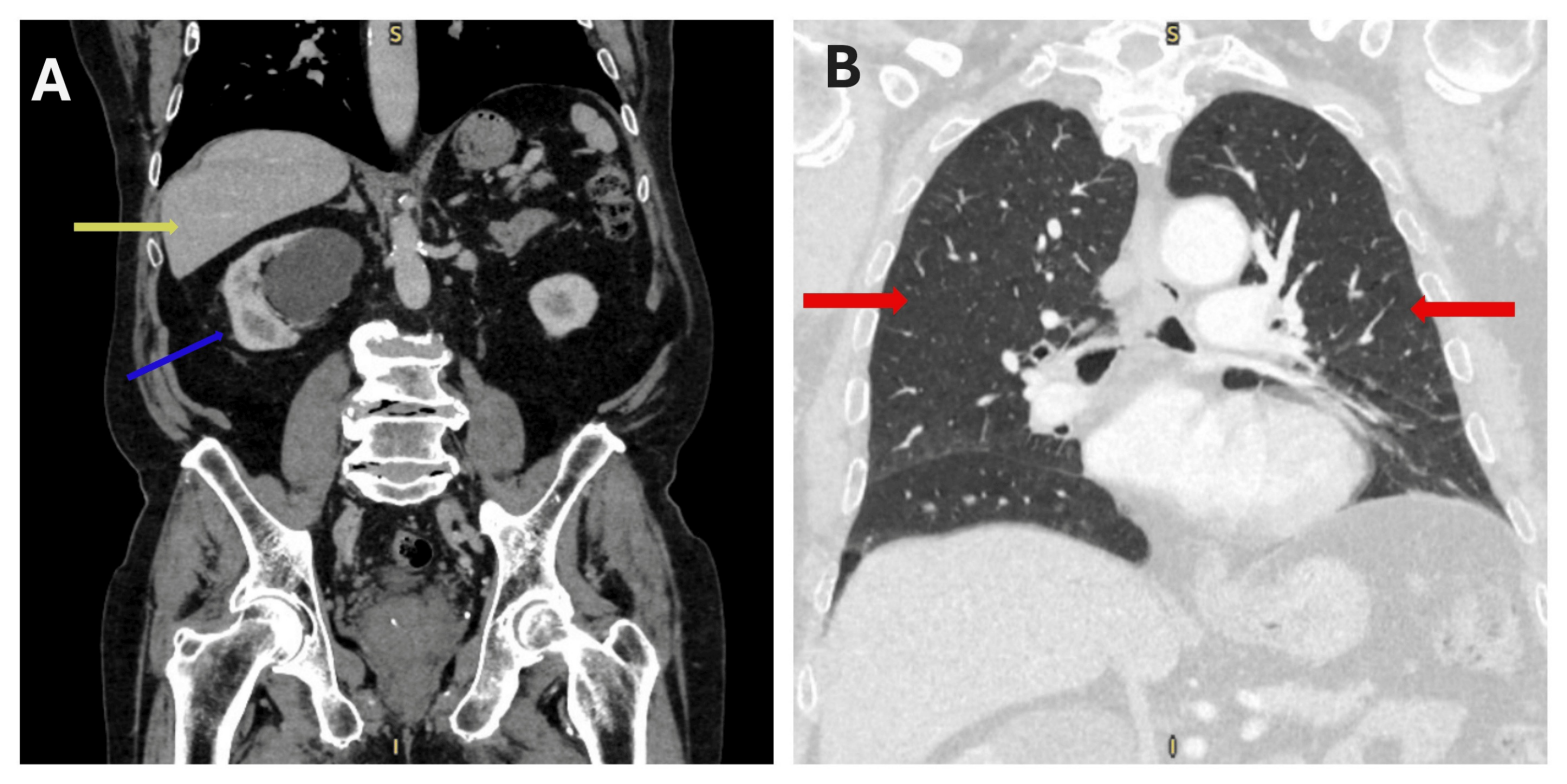
Postoperative staging CT scan of the thorax, abdomen, and pelvis with contrast. (A) Abdomen and pelvis showing no metastasis. The yellow arrow points to the liver and the blue arrow points to the right kidney with an incidental simple cyst. (B) Chest scan showing no metastasis (red arrows point to the lungs).

Therefore, after multidisciplinary team meeting (MDT) discussion, it was agreed that a radical inguinal orchidectomy and a staging CT scan of the thorax, abdomen, and pelvis with contrast are warranted. Orchidectomy was performed, and radiological staging with CT scan revealed no evidence of metastasis (Figure 8).

**Figure 9:**
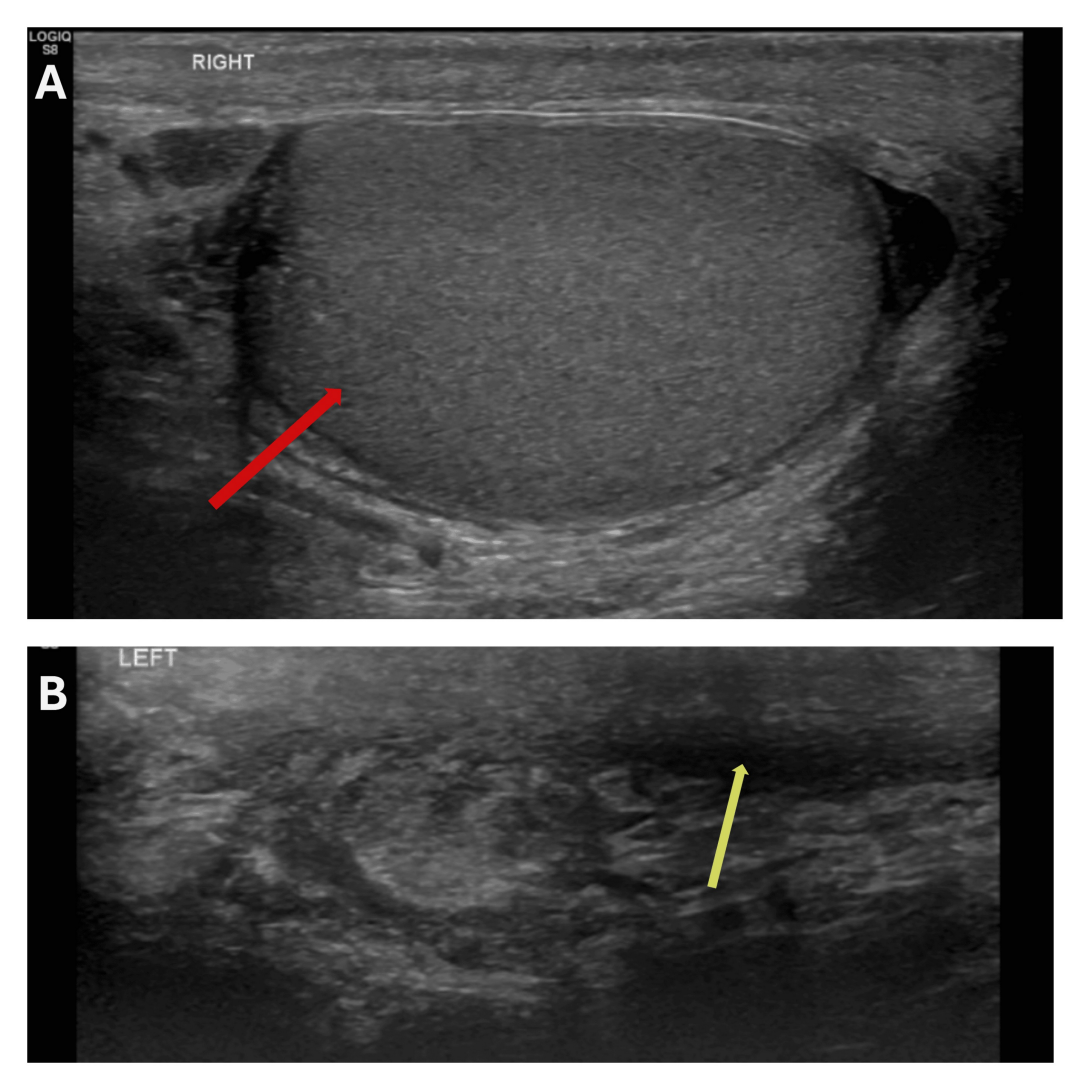
Six-month follow-up scrotal ultrasound showing (A) a normal right testicle (red arrow) and (B) a left empty hemiscrotum with no local recurrence (yellow arrow).

**Figure 10:**
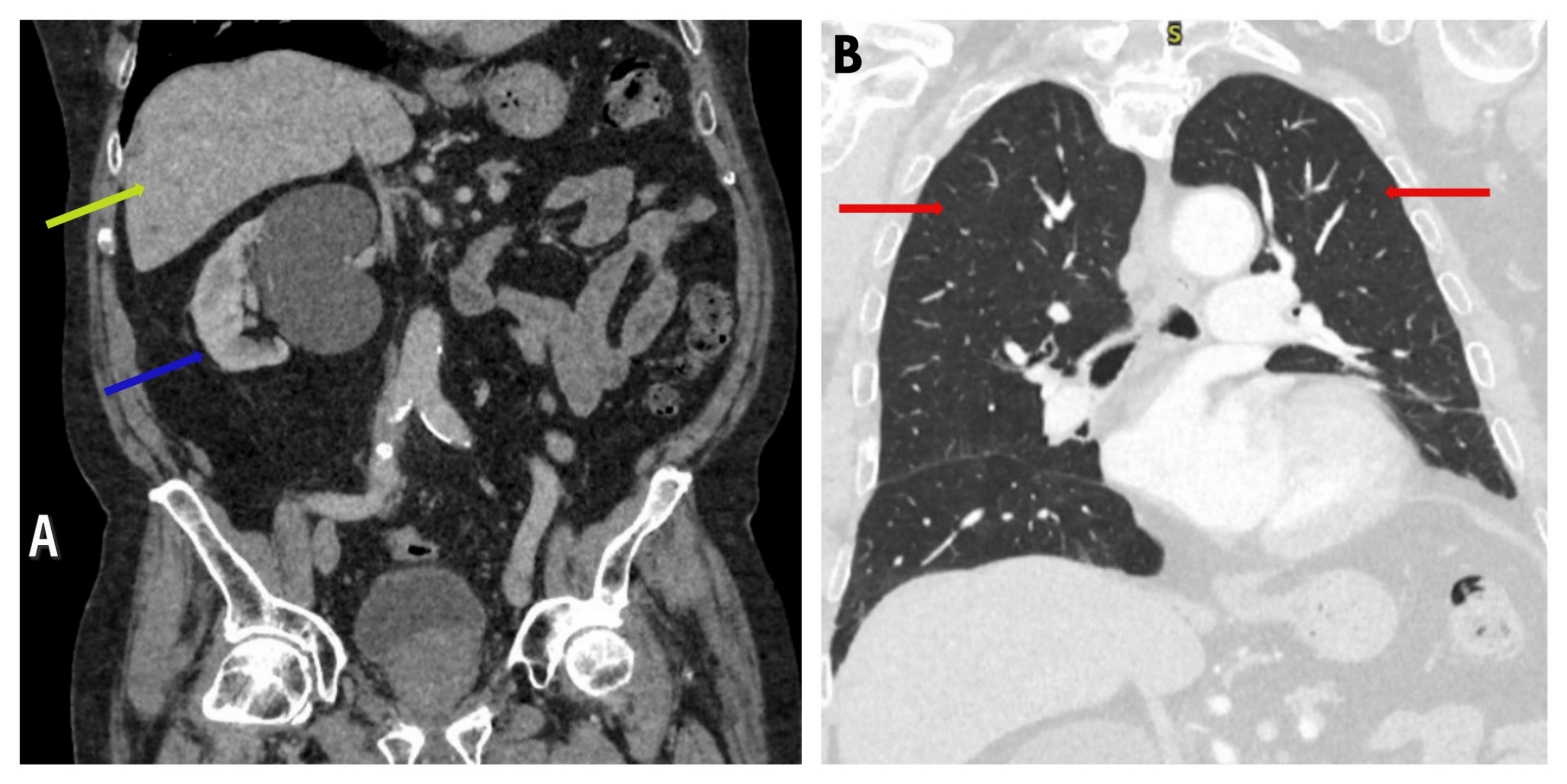
Six-month follow-up contrast-enhanced CT scan of the thorax, abdomen, and pelvis. (A) Abdomen and pelvis showing no metastasis. The yellow arrow indicates the liver, and the blue arrow indicates the right kidney with a simple cyst. (B) Chest scan showing no metastasis (red arrows point to the lungs).

The plan was made for the patient to have close follow-up with scrotal ultrasound, given the high risk of local recurrence, and CT scan of the thorax, abdomen, and pelvis to monitor for metastatic disease. At the six-month follow-up, scrotal ultrasound showed no evidence of local recurrence (Figure 9), and CT scan showed no evidence of metastasis (Figure 10).

## Discussion

The disease in our patient, an elderly man with a large appearing benign hydrocele at presentation, which was diagnosed only post-hydrocelectomy and confirmed on radical inguinal orchiectomy, follows the typical diagnostic course as described from prior experience of this tumour [2,6,7,12]. The immunohistochemistry showed a typical mesothelial profile (positive for calretinin, WT-1, D2-40; negative for CEA, Ber-Ep4), as described in large series and emphasizing the value of a broad mesothelial panel and directed carcinoma-exclusion markers in avoiding diagnosis pitfalls in paratesticular disease [8-11]. No metastatic disease has been detected on perioperative staging scans, and the patient had no evidence of disease recurrence or metastasis at short-interval follow-up, which we realize we can expect for this subset of cases that present with organ-confined disease.

In terms of epidemiology, MTVT generally develops in men in the sixth to eighth decades, consistent with our case [1,2]. The contribution of asbestos to aetiology is uncertain: Italian registry-based reporting and review data indicate weak or absent associations for MTVT compared with pleural disease, whereas aggregates of case series consistently demonstrate a significant minority with suggested exposure [1,4,5]. Mesothelial multiplication and malignant transformation can develop due to factors other than asbestos exposure, most notably chronic serosal inflammation (chronic hydrocele, recurrent infections, prior scrotal operations); however, proof of a causal association is limited [1,2].

Imaging clues can be subtle, as in our case. The most specific sonographic pattern is a complex hydrocele with hypervascular parietal vegetations when visible; however, one-third of cases present with a nonspecific solid paratesticular mass or “simple” hydroceles, which may hide MTVT, and surgery is therefore delayed for those patients [6,7]. As a result, some authors advocate low thresholds for histological examination of the hydrocele walls or any suspicious intratunical nodularity at the time of hydrocelectomy.

The treatment of choice, in the past as well as at present, is radical inguinal orchiectomy. Series from the NIH and other centres now establish that total resection with negative margins is the single most important prognostic factor, with positive margins indicating a significantly worse survival and increased risk of clinical relapse [12,13]. Case series have reported selective hemiscrotectomy and regional lymphadenectomy, especially in the setting of scrotal depilation, dense adhesion, or nodal disease; little has been proven to be of clear benefit, as only limited numbers [12,13]. The history of some early pooled analyses also suggested recurrence rates of 60 % within the first two years, emphasising the need for coordinated clinic follow-up with early cross-sectional imaging [16]. Our patient is currently under six-monthly scans and clinic follow-up.

There is sparse information regarding systemic therapy for MTVT in particular. Platinum-pemetrexed is borrowed from the management of pleural mesothelioma, where it is associated with a survival advantage over cisplatin alone; gemcitabine-carboplatin is an alternative on the basis of phase II activity in pleural disease [14,15]. More recently, inhibitors of PD-1 and PDL-1 (and CTLA-4, i.e., nivolumab-ipilimumab) have shown activity in pleural mesothelioma and at least one confirmed partial response in MTVT, implying that the latter may represent a reasonable off-label choice in a subset of patients who have failed standard therapies [17,18]. Prospective evaluation in MTVT is less likely (although numbers of well-documented cases are increasing). High-quality documentation of individual cases may be a stimulus to enhance patient selection and improve genetic counselling.

In summary, this case brings up several salient points: (i) MTVT should be in the differential diagnosis for older adult men presenting with atypical or recurrent hydroceles; (ii) submission of hydrocele walls and intratunical excrescences for histology can yield a diagnosis; (iii) mesothelial IHC panels are essential to differentiate MTVT from its morphological mimics; and (iv) radical inguinal orchiectomy with negative margins is the primary treatment modality, and close follow-up is important as there is a risk of early recurrence [6,8-13,16].

## Conclusions

Malignant mesothelioma of the tunica vaginalis testis remains an extremely rare malignancy that often presents as a benign-appearing hydrocele, resulting in a delay in diagnosis or misdiagnosis. This case emphasizes the diagnostic utility of routine histological examination for atypical or recurrent hydroceles and the key role of immunohistochemistry in establishing mesothelial origin and excluding mimics. Radical inguinal orchiectomy with clear resection margins, which can be curative, is the treatment of choice with the highest potential of local disease control; however, the risk of relapse is not insignificant, and long-term prognosis is relatively poor. In the absence of standardised guidance, close follow-up is paramount, and systematic reporting of these cases is necessary to accumulate cumulative evidence, enable us to refine our management, and optimize future patient outcomes.
